# Supplementary material for: No effect of repetitive tDCS on daily smoking behaviour in light smokers: A placebo controlled EMA study
Source: PLoS One. 2020 May 22;15(5):e0233414. doi: 10.1371/journal.pone.0233414 (PMC7244103; doi:10.1371/journal.pone.0233414)
Supplement: Attachment — Submitted filename: Thank you5.docx [file pone.0233414.s006.docx]

Dear Editor

Thank you for the opportunity to review this manuscript. 

This is a study that seeks to determine the effect of tDCS on smoking behavior. Overall, the topic is important; however, a number of issues may limit the value of the article, I hope below comments improve the quality of the paper.

**Title**

According to the CONSORT statement, authors should indentify in the title that this is a controlled trial. Abbreviated words also should be removed from the title.

**Abstract**

1. Introduction is too long. Here the authors should use effectiveness instead of efficacy.

The sentence “To determine the duration of treatment effects, daily smoking behavior was studied for three months.” should be moved to method section.

2. The definition of all outcomes and statistical methods should be provided.

3. In contrast to introduction with unnecessary sentences, the results section is very brief with no effect sizes, confidence intervals and P values!

3. The conclusion should be according to presented results.

**Introduction**

1 Line 27, please provide the latest evidences

2. Lines 33-34, Please provide references for this sentence.

3. Line 44, marihuana or marijuana?

4. Line 46-52. There are still other related studies which should be cited such as:

https://www.sciencedirect.com/science/article/pii/S0924933819300793

5. Lines 56-63. Current study is also with limited sample size and follow up for three months is not long follow up, therefore it is not clear that what will be added this study to current knowledge.

6. I am not agreeing with authors that previous studies used **retrospective measurements!** with some degree of recall bias. Especially EMA is a subjective measurement with high probably of information bias.

**Methods**

1. No information provided about randomization type and method, concealment method, sample size calculation. Please consider CONSORT with caution.

2. The information about blinding and statistical methods is also insufficient. I am not believed that this study is double blind.

3. More details are required about assessments (i.e RA, SA and EA).

4. Line 121, why the results of behavioral tasks are beyond the scope of this paper?! Authors should provide all outcomes, declared in clinicaltrials.gov registration.

5. I am not sure that the method used for measurement of craving and mood are correct and based on DSM5.

6. Simple statistical methods such as independent t-test for changes over different intervals, repeated measured ANOVA and GEE are more appropriate especially for general readers. The methods for comparing baseline data should also be provided.

**Results**

1. It is unusual that with including participants who smoked more than 10 cigarettes per day, the mean daily cigarettes smoked become 11.29 with a SD equal to 4.2. Please provide the range of cigarettes smoked per day.

2. Line 194-198, with a true randomization there is no need for matching. Is this a randomized control trial?? The differences in age and craving of participants are attributed to inappropriate or lack of randomization although small differences between baseline variables may be present due to random error. Again please consider CONSORT and compare all studied variables at the baseline and different intervals.

3. The compliance rates for RA and EA are very low and this is a big limitation. Surprisingly authors did not compare these rates between intervention and control groups. Non respondents should be compare according to the other outcomes.

4. There are many fluctuations in number of smoked cigarettes in both groups (Fig 3). When one group show increased number of cigarettes other groups show decreased smoked cigarettes! This may be attributed to incorrect randomization, low compliance rate and information bias.

5. The results of statistical test should be provided in tables with all details.

6. Although the P-values are significant, the correlation coefficients between smoked cigarettes and CO are below 0.5 and contrary to the authors' claim it shows a low correlation.

**Discussion**

1. Lines 270-271, I am not agree that the data collected by EMA is more valid than previous studies. This study has many methodological errors.

2. Lines 282-283, Low compliance rate is an important issue. By excluding participants with low compliance, the sample size will be decrease (Although the total sample size was also low) and lack of significant difference between groups can be attributed to type two error.

3. Discussion should be revised after new and complete analysis of data and with addressing many limitations of study.

**References**

Should be updated.

**Figures**

Have low resolution and should be revised according to the new results.
