# Supplementary material for: No effect of repetitive tDCS on daily smoking behaviour in light smokers: A placebo controlled EMA study
Source: PLoS One. 2020 May 22;15(5):e0233414. doi: 10.1371/journal.pone.0233414 (PMC7244103; doi:10.1371/journal.pone.0233414)
Supplement: Attachment — Submitted filename: Appeal request incl modifications PONE-D-19-19431.docx [file pone.0233414.s007.docx]

Dear Editor,

Thank you for your time and effort to review our resubmitted manuscript entitled “Effects of Repetitive tDCS on ad libitum Smoking Behavior: An EMA Study”. We are certain that the manuscript has previously been taken into careful consideration by expert reviewers in our field. And one of us (I.F.) - having been an editor of PLOS ONE for many years – is very aware that editoral decisions are not always easy. That being said, we were a bit surprised that our paper was rejected the first time by PLOS ONE since two out of three reviewers were quite positive and stated that we used an adequate methodology. The third reviewer was more critical but we believe that several responses of particularly this reviewer seem to indicate that there was some misconception about important aspects of the method and result section which we addressed in this appeal letter. In addition, we feel it is important that also null findings are published, particularly in journals such as PLOS ONE.

Considering all responses, the following seem crucial for the rejection of the current paper:

1. Participants were light smokers:

This is correct, we were not only interested in heavy smokers, but all (including light) smokers. We have addressed this more clearly in the resubmitted paper.

2. Motivation to quit has not been taken into account:

We did not take motivation into account since it only became apparent that motivation may influence the effects of tDCS after we had started the data collection. That is, the data collection started end of 2016 and the paper which indicates that motivation to quit may be an important modulation factor appeared in 2018. We therefore mention in our discussion that this might be a valid explanation for our null-findings.

3. The sham and active tDCS group differed in craving at baseline:

This is an unfortunate result of our double-blind randomization procedure. To our surprise, the reviewer in question (3) mentions that “he/she is not believed that the study is double-blind”. In appeal to this response, we would like to refer to lines 141-143 where it’s stated that the tDCS system enabled the researcher to fill out a code which starts the procedure (either sham or active tDCS). The codes were assigned to participant numbers by an independent researcher. The procedure was therefore most certainly double-blind. We therefore believe that this baseline difference is the unfortunate result of chance.

4. Compliance rate of EMA responses was low:

This is correct and we agree. However, as mentioned by our review paper on this topic (Jones and colleagues; line 287 – 289) little is known about how to increase compliance rates. To correct for the low compliance rate, we performed exploratory analysis only including participants with a high compliance rate. This did not result in different outcomes. Other indicators of the reliability of our EMA data are also mentioned (line 290-298).

Point-by-Point responses to the reviewer:

**Title and Abstract**

The title and point 1-4 for the abstract have been adjusted in revisions.

**Introduction**

Point 1-3: Indicated that small adjustments had to be made in certain sentences.

Point 4: The reviewer referred that the following paper should have been cited: <https://www.sciencedirect.com/science/article/pii/S0924933819300793>. However, this article was published in August 2019, whereas we submitted the manuscript in July. We have included the reference in the revised version of our manuscript.

Point 5: The reviewer mentioned that the study has a limited sample size and that 3 months is not a long follow-up. Still, the sample size is larger compared to previous studies with a between subject design that investigated the effects of tDCS on smoking behavior.

As far as we know, the largest sample for active tDCS in smokers was 19 participants (<https://www.ncbi.nlm.nih.gov/pmc/articles/PMC5791546/pdf/fphar-09-00014.pdf>) before we submitted the manuscript in Juli 2019, whereas we’ve included 35 participants for active stimulation. In addition, 3 months follow-up was the longest follow-up that had been performed in smokers so far.

Point 6: The reviewer claimed that previous studies did not use retrospective self-reports. However, in comparison with ecological momentary assessments by which smoking behavior multiple is measured multiple times a day in different contexts, questionnaires at the end of the day or week (in the lab) are retrospective and could therefore affect recall. That is, since for example craving is a momentary phenomenon, and this sensation can be difficult to recall after a while. Participants also indicated that they became aware of how often they smoked by filling in the app, and that this was often more than they thought in retrospect.

**Method:**

Points 1 and 2: We would like to refer to line 104-106 and line 143-145, and the response above stating that our study design was randomized and double-blind.

Point 3: Details about assessments are provided under “Procedure” and “Measures”.

Point 4: Behavioral tasks are beyond the scope of the paper because these also include EEG measures. It would make the article unnecessarily long and incomprehensive, and therefore we decided to publish the outcomes of the tasks in a separate article.

Point 5: The reviewer mentions that simpler statistical methods could have been used. This is not the case since our data has a multilevel design with Time within participants at level 1 and Group at level 2, and should therefore be analyzed as such. We have addressed this more clearly in our revision (lines 176-182).

**Results:**

Point1: Participants were indeed lighter smokers. We have addressed this in the discussion (Line 300).

Point2 and 3: Randomization and compliance are addressed above.

Point 4: The reviewer mentions that many fluctuations in number of smoked cigarettes can be attributed to flaws in the research design. However, it’s more likely that smoking and craving (at least in light smokers) fluctuates over time according to mood and context. At the same time, this result therefore indicates the importance of using EMA measures.

All other points are related to above mentioned points or were small points that have been taken into account in the revision.

All in all, we don’t agree that there are flaws in the research design and/or methodology and therefore sincerely hope that you can find the time to take our resubmitted manuscript into consideration.

Yours Sincerely,

Ingmar Franken and Ilse Verveer
